# Evaluation of computed tomography in the diagnosis of ultrasound-proven diaphragm dysfunction

**DOI:** 10.1186/s12931-024-02770-w

**Published:** 2024-03-20

**Authors:** Pauline Lallement, Alain Boussuges, Paul Habert, Julien Bermudez, Martine Reynaud-Gaubert, Stéphane Delliaux, Fabienne Bregeon, Benjamin Coiffard

**Affiliations:** 1 Department of Respiratory Medicine and Lung Transplantation, Aix Marseille University, APHM, Chemin des Bourrely, 13015 Marseille, France; 2 Pulmonary Function Testing Laboratory, Aix-Marseille University, APHM, Marseille, France; 3 Department of Radiology, Aix-Marseille University, APHM, Marseille, France; 4 LIIE, Aix Marseille University, Marseille, France; 5 CERIMED, Aix Marseille University, Marseille, France

**Keywords:** Diaphragm, Ultrasonography, X-Ray computed tomography, Physiology, Respiratory physiological phenomena, Musculoskeletal physiological phenomena, Respiratory function tests

## Abstract

**Introduction:**

Computed tomography (CT) is routinely employed on the evaluation of dyspnea, yet limited data exist on its assessment of diaphragmatic muscle. This study aimed to determine the capability of CT in identifying structural changes in the diaphragm among patients with ultrasound-confirmed diaphragmatic dysfunction.

**Methods:**

Diaphragmatic ultrasounds conducted between 2018 and 2021 at our center in Marseille, France, were retrospectively collected. Diaphragmatic pillars were measured on CT scans at the L1 level and the celiac artery. Additionally, the difference in height between the two diaphragmatic domes in both diaphragmatic dysfunction cases and controls was measured and compared.

**Results:**

A total of 65 patients were included, comprising 24 with diaphragmatic paralysis, 13 with diaphragmatic weakness, and 28 controls. In the case group (paralysis and weakness) with left dysfunctions (*n* = 24), the CT thickness of the pillars at the level of L1 and the celiac artery was significantly thinner compared with controls (2.0 mm vs. 7.4 mm and 1.8 mm vs. 3.1 mm, *p* < 0.001 respectively). Significantly different values were observed for paralysis (but not weakness) in the right dysfunction subgroup (*n* = 15) (2.6 mm vs. 7.4 mm and 2.2 mm vs. 3.8 mm, *p* < 0.001 respectively, for paralysis vs. controls). Regardless of the side of dysfunction, a significant difference in diaphragmatic height was observed between cases and controls (7.70 cm vs. 1.16 cm and 5.51 cm vs. 1.16 cm, *p* < 0.001 for right and left dysfunctions, respectively). Threshold values determined through ROC curve analyses for height differences between the two diaphragmatic domes, indicative of paralysis or weakness in the right dysfunctions, were 4.44 cm and 3.51 cm, respectively. Similarly for left dysfunctions, the thresholds were 2.70 cm and 2.48 cm, respectively, demonstrating good performance (aera under the curve of 1.00, 1.00, 0.98, and 0.79, respectively).

**Conclusion:**

In cases of left diaphragmatic dysfunction, as well as in paralysis associated with right diaphragmatic dysfunction, CT revealed thinner pillars. Additionally, a notable increase in the difference in diaphragmatic height demonstrated a strong potential to identify diaphragmatic dysfunction, with specific threshold values.

**Supplementary Information:**

The online version contains supplementary material available at 10.1186/s12931-024-02770-w.

## Introduction

Diaphragmatic dysfunction (DD) involves a diminished diaphragm motion, which can manifest as either complete (paralysis) or partial (weakness). Currently, this condition is often underdiagnosed and should be consistently contemplated as a potential differential diagnosis in cases of unexplained breathlessness. DD has the potential to result in restrictive ventilatory disorders, dyspnea, sleep disturbances, atelectasis, and, in severe cases, chronic respiratory failure [1].

Various validated methods exist for diagnosing DD, but they are not universally accessible and may have inherent limitations [2–6]. Among the available techniques, diaphragmatic ultrasound stands out as a relatively easier method. This rapid, non-invasive, and reproducible examination, while having some limitations (operator-dependence, position-dependence, and challenges in visualizing the diaphragm, particularly on the left side and in obese patients) [7, 8].

Notably, thoracic computed tomography (CT) is not yet a widely adopted method for DD diagnosis, despite being frequently in the diagnostic evaluation of dyspnea. In comparison to diaphragmatic ultrasonography, CT offers a comprehensive depiction of diaphragmatic anatomy [9], is more widely available, not operator-dependent, and reproducible. Previous studies have demonstrated that in cases of unilateral diaphragmatic paralysis, CT can reveal thinning of the diaphragmatic pillars, with measurements feasible at the at the level of the celiac artery and L1 [10]. Furthermore, the measurement of pillar thickness at these locations has shown good intra- and inter-observer reproducibility [11].

We postulated that CT could contribute to the diagnostic evaluation of DD characterized by paralysis or weakness. The primary objective of this exploratory study was to investigate the potential of CT in detecting structural alterations in the diaphragm among patients with confirmed DD as determined by ultrasound examination.

## Methods

### Study population

This retrospective observational study was conducted at North Hospital, Marseille, France. The study encompassed all patients who underwent diaphragmatic ultrasound examinations at our center between 2018 and 2021. Classification into three groups − paralysis, weakness, or controls (absence of DD) − was based on the findings of the diaphragm ultrasound. Patients without a concurrent CT scan (utilized in this study for the assessment of diaphragmatic pillars), as part of their respiratory functional exploration, were excluded from the analysis.

Clinical data including age, sex, height, weight, body mass index (BMI), blood albumin levels, cause of DD, and patient medical history, were systematically extracted from the medical charts for comprehensive analysis.

The Institutional Review Board of the French Learned Society for Respiratory Medicine, Société de Pneumologie de Langue Française, granted approval for the study protocol under the reference CEPRO 2022–043. All participants were provided with a notice of information and non-objection in compliance witch French law. The study strictly adhered to the ethical standards outlined in the 2008 Declaration of Helsinki.

### Ultrasound evaluation

Diaphragm ultrasound measurements were conducted with patients in a seated position, assessing both right and left side using the same ultrasound device (Vivid S60N, GE Healthcare, Milwaukee, Wl, USA). All measurements, encompassing both cases and controls, were consistently executed by a singular experienced operator (AB). In order to enhance result precision, each measurement was derived from an average of at least three distinct breathing cycles.

The diaphragm excursions (amplitudes) were investigated in M-mode, utilizing a cardiac probe (3Sc probe), during different respiratory maneuvers, including quiet breathing (QB), voluntary sniffing (VS), and deep breathing (DB), as per a previously published methodology [12]. Additionally, diaphragmatic thickness at end-expiration and maximal thickening fraction (TF_max_) were assessed in B-mode using a linear vascular transducer (9L probe), following a previously established protocol [13].

Diaphragmatic weakness was characterized by an amplitude during DB falling below the normal range. Severe weakness was identified by an amplitude during DB below normal and a TF_max_ less than 40% [13, 14]. The established lower limits for diaphragmatic amplitude during DB were 3.3 cm in women and 4.1 cm in men on the right side, and 3.2 cm in women and 4.2 cm in men on the left side [15].

Diaphragmatic paralysis was defined by a paradoxical movement during VS (cranial movement of the paralyzed dome) and at the initiation of deep inspiration without inspiratory thickening (TF_max_ < 20%) [5, 12, 16]. In instances of quiet breathing, movement of the paralyzed dome may be absent or paradoxical [17, 18].

### CT scan evaluation

The review of CT scans were conducted by a chest radiologist (PH). All thoracic CTs were executed during the inspiratory phase, adhering to the specified parameters: 120 kV and 1 mAs/kg with care dose modulation, and reconstruction in isotropic slices of 1:1 mm. Dose adjustments were manually based on the patient template: 100 kV for individuals weighing less than 60 kg and above 120 kV for others. Thoracic CT scans were acquired during breath-hold inspiration, spanning from the adrenal glands to the neck. Various systems, including Revolution EVO, Revolution Frontier, and Revolution CT from GE Healthcare, WI, USA, were employed for the CT scans.

**Fig. 1:**
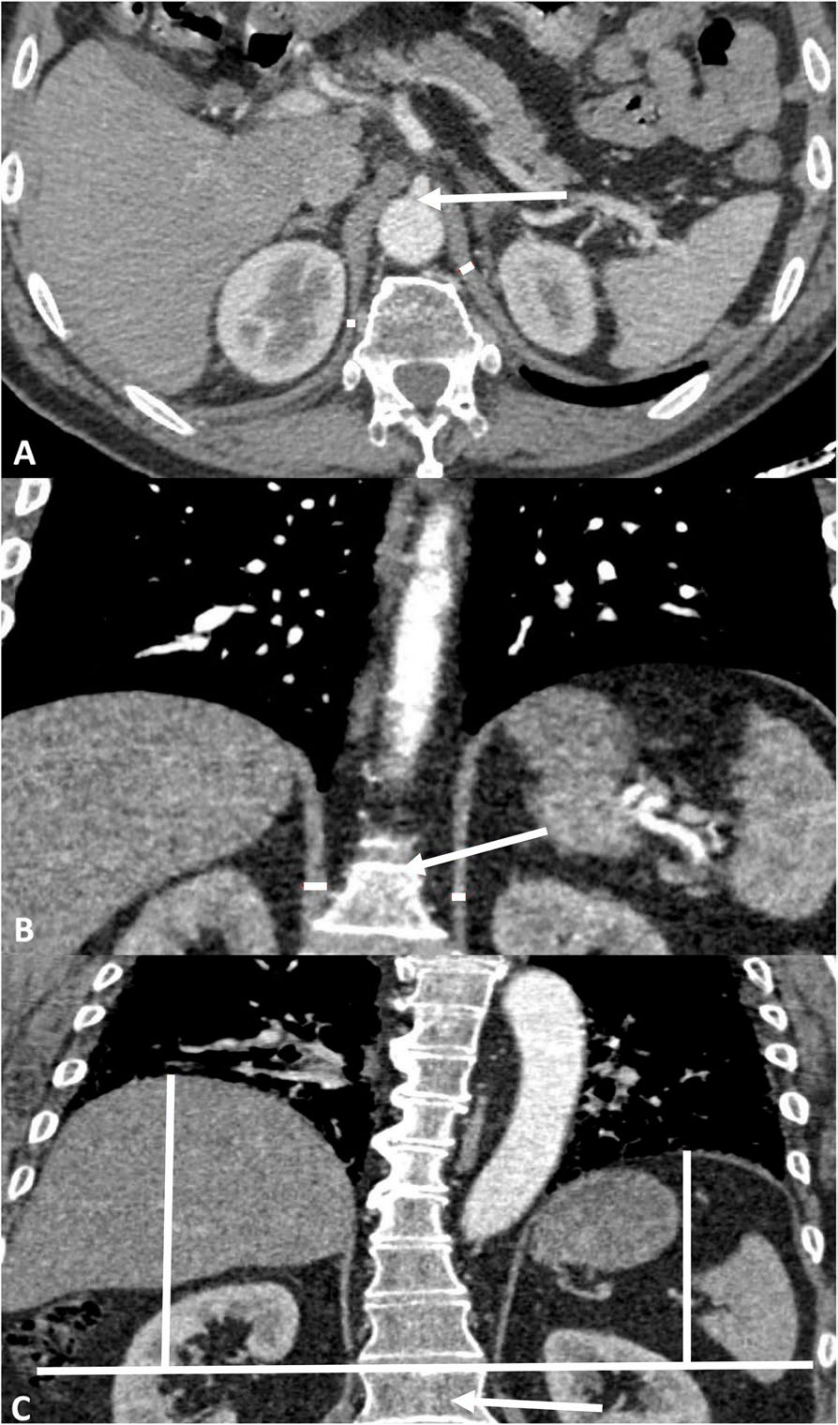
Measurements of the diaphragm performed on enhanced CT scan. **A** Measurement of the pillars at the level of the celiac artery. Minimal diaphragm thicknesses (red lines) at the level of the origin of the celiac artery (red arrow). **B** Measurement of the pillars at the level of L1. Diaphragm thicknesses (red lines) along the anterior part of the L1 vertebral body at mid-level (red arrow). **C** Measurement of the diaphragm height difference. Heights (red lines) from the highest point of the diaphragmatic dome to its perpendicular intersection with a line (white line) following the upper plateau of L1 (red arrow)

Diaphragmatic pillars were assessed in both axial and coronal images. In axial images, the right and left pillars were measured at the level of the celiac artery origin, and the minimal thickness was documented (Fig. 1A). On coronal images, measurements of the right and left pillars were taken along the middle of the anterior part of L1 (Fig. 1B), aligning to the methodology established by Sukkasem et al. [10]. Additionally, on coronal images, the disparity in height between the two diaphragmatic domes was measured at the anterior part of L1 (Fig. 1C).

### Statistics

Continuous variables were presented as mean and standard deviation or median and interquartile range, depending on their distribution (Shapiro test). Categorical variables were described in terms of proportions. Group comparisons between the control and DD groups were conducted using the Chi-2 test for categorical variables. For continuous variables, either Student's t-test or the non-parametric Mann–Whitney-Wilcoxon test was applied, depending on the distribution. Receiver Operating Characteristic (ROC) curves were constructed to evaluate the probability of DD presence based on CT measurements, with ultrasound serving as the reference standard for the diagnosis of DD. Area under the curve (AUC) values were calculated, along with the diagnostic performance metrics for each CT measurement, including sensitivity, specificity, positive and negative predictive value. The optimal diagnostic thresholds were determined by the ROC curves.

All tests were two-sided, and statistical significance was defined as a p-value < 0.05. The analysis was conducted using R software version 4.2.1 (2022–06-23) (R Core Team (2018). R: A language and environment for statistical computing. R Foundation for Statistical Computing, Vienna, Austria. URL https://www.R-project.org/).

## Results

We identified a total of 92 diaphragmatic ultrasounds conducted during the study period. Of these, 27 were excluded due to the unavailability of a concomitant CT scan (21 cases of paralysis, 3 cases of weakness, and 3 control subjects). The remaining 65 patients were included in the analysis as they had undergone a concurrent thoracic or abdominal CT scan, facilitating the measurement of diaphragmatic parameters. The median time lapse between CT scan and diaphragmatic ultrasound for the entire population was -13 days, with 1st and 3rd quartiles ranging from -49 to 23 days. Within the subset of 65 patients, 24 exhibited diaphragmatic paralysis (7 on the right, 16 on the left, and 1 bilateral), 13 showed diaphragmatic weakness (6 on the right, 6 on left, and 1 bilateral), and 28 had normal diaphragmatic ultrasound.

The detailed characteristics of the patients included in the study are provided in Table 1. The primary cause of diaphragmatic paralysis was identified as post-surgical, whereas post-COVID-19 was identified as the main cause of diaphragmatic weakness. Controls consisted of individuals post-COVID-19 who sought evaluation for dyspnea without significant medical history. No statistically significant differences were observed in terms of age, sex, height, BMI, or duration of intensive care stay between the DD group and the control group.

**Table 1:** Clinical and functional characteristics of the patients included in the study

	Dysfunctions	Controls	p	Paralysis	Weakness	p
**Patients, n (%)**	37 (57)	28 (43)		24 (37)	13 (20)	
**Side, n (%)**						
*Right*	13 (35)			7 (30)	6 (46)	0.48
*Left*	22 (60)			16 (67)	6 (46)	
*Bilateral*	2 (5)			1 (4)	1 (8)	
**Cause**						
*Traumae*	8 (21)	0 (0)		6 (25)	2 (15)	0.001
*Surgery*	13 (35)	0 (0)		13 (54)	0 (0)	
*Cancer*	2 (5)	0 (0)		2 (8)	0 (0)	
*Parsonage Turner*	1 (3)	0 (0)		0 (0)	1 (8)	
*Eventration*	1 (3)	0 (0)		1 (4)	0 (0)	
*COVID-19*	7 (19)	28 (100)		1 (4)	6 (46)	
*Pneumonia*	1 (3)	0 (0)		0 (0)	1 (8)	
*Idiopathic*	4 (11)	0 (0)		1 (4)	3 (23)	
**ICU stay**	6 (16)	7 (28)	0.57	2 (8)	4 (31)	0.19
*MV*	6 (16)	4 (14)	1	2 (8)	4 (31)	0.19
*Length of MV*	24 [16; 83]	16 [12; 19]	0.78	17 [10; 25]	58 [16; 100]	0.07
**Age**	60 [47; 73]	56 [54; 60]	0.29	62 [47; 71]	60 [51; 73]	0.75
**Sex (Women)**	18 (49)	10 (36)	0.32	17 (71)	1 (8)	< 0.001
**Height**	1.66 [1.60; 1.78]	1.69 [1.65; 1.75]	0.85	1.64 [1.59; 1.69]	1.78 [1.67; 1.80]	0.012
**Weight**	74 ± 20	81 ± 19	0.22	66 ± 17	89 ± 16	< 0.001
**BMI**	26 ± 6	28 ± 6	0.14	24 ± 5	30 ± 5	0.004
**Albumin**	43 [39; 46]	45 [41; 47]	0.23	43 [38; 46]	42 [41; 45]	0.69
**PFT**						
*FVC (L)*	2.35 [1.91; 2.88]	4.05 [3.23; 4.68]	< 0.001	2.34 [1.91; 2.92]	2.35 [1.97; 2.80]	0.75
*FVC (%pred)*	69 ± 17	100 ± 14	< 0.001	76 ± 14	56 ± 12	< 0.001
*FEV1 (L)*	1.84 [1.45; 2.08]	3.32 [2.68; 3.74]	< 0.001	1.75 [1.34; 2.17]	1.97 [1.62; 2.08]	0.48
*FEV1 (%pred)*	65 [55; 72]	104 [96; 112]	< 0.001	70 [61; 82]	56 [55; 62]	0.02
*Tiffeneau*	78 ± 11	83 ± 5	0.045	76 ± 12	82 ± 7	0.14
*TLC (L)*	4.7 ± 1.2	6.1 ± 1.3	0.06	4.9 ± 1.2	4.1 ± 1.0	0.06
*TLC (%pred)*	85 [69; 97]	100 [93; 105]	< 0.001	94 [81; 98]	66 [56; 70]	< 0.001
**Respiratory pattern**						
*None*	6 (16)	26 (93)	< 0.001	5 (21)	1 (8)	0.17
*Restrictive*	26 (70)	2 (7)		14 (58)	12 (92)	
*Obstructive*	2 (6)	0 (0)		2 (8)	0 (0)	
*Mixte*	3 (8)	0 (0)		3 (12)	0 (0)	
**Time between CT and US**	58 [20; 128]	23 [14; 32]	0.32	65 [18; 134]	52 [31; 66]	0.31

*BMI* body mass index, *ICU* intensive care unit, *PFT* pulmonary function test, *FVC* forced vital capacity, *FEV1* forced expiratory volume in 1 s, *Tiffeneau* FEV1/CV, *MV* mechanical ventilation, *TLC* total lung capacity

Within the paralysis group, there was a higher representation of women compared to the weakness group (71% versus 8%, *p* < 0.001), and the mean BMI was lower (24 versus 30 kg/m2 in the weakness group, p = 0.004). Sixty percent of patients exhibited left-sided DD, 35% right-sided, and 5% bilateral. The distribution of paralysis and weakness did not show significant differences based on the affected side (p = 0.48). In the DD groups, patients with weakness demonstrated lower Forced Vital Capacity (FVC) in percentage predicted (mean of 56% versus 76% in the paralysis group, *p* < 0.001) and lower Total Lung Capacity (TLC) in percentage predicted (mean of 66% versus 94% in the paralysis group, *p* < 0.001). However, the presence of obstructive or restrictive patterns did not significantly differ between the paralysis and weakness groups (*p* = 0.17).

The diaphragmatic measurements obtained from ultrasound and CT scans are summarized in Table 2. On ultrasound, for both right and left DD, all measurements were significantly lower between in cases compared to controls, except for end-expiratory thickness in weakness cases, which did not differ significantly from controls. On CT scan, in the presence of left DD, both the thickness of the pillars at L1 (*p* < 0.001) and the celiac artery (*p* < 0.001) were significantly lower compared with controls. In right DD, thicknesses of paralyzed patients (and not weakness) were lower (*p* < 0.001 at the L1 level and *p* = 0.004 at the celiac artery level). For both right and left DD, the difference in median height between the two domes was significantly greater in the DD group compared to controls (*p* < 0.001 for both sides).

**Table 2:** Analyses of ultrasound and CT scan measurements according to cases and controls

				Para. vs. Weak		DD Vs. Controls	Para. Vs. Controls	Weak. Vs. Controls		Contral. DD Vs. Controls
Right dysfunction	DD	Paralysis	Weakness	*p*	Controls	*p*	*p*	*p*	Contralateral	*p*
**N (%)**	15 (23)	8 (12)	7 (9)		28 (43)				13 (20)	
**Ultrasound**										
*Amplitude during QB (cm)*	0.90 [0.15; 1.43]	0.30 [-0.03; 0.84]	1.35 [1.05; 1.73]	0.02	2.00 [1.72; 2.37]	< 0.001	< 0.001	0.01	2.90 [2.60; 3.36]	0.005
*Amplitude during DB (cm)*	1.75 [-0.30; 2.76]	-0.30 [-0.43; 1.85]	2.45 [1.85; 3.13]	0.04	5.69 [5.23; 5.93]	< 0.001	< 0.001	< 0.001	5.28 [4.95; 5.62]	0.20
*Amplitude during VS (cm)*	-0.04 [-1.17; 1.37]	-1.15 [-1.30; -0.90]	1.50 [1.33; 1.90]	0.003	2.37 [1.96; 3.07]	< 0.001	< 0.001	0.004	3.30 [2.70; 3.56]	0.02
*Tee (mm)*	1.67 ± 0.49	1.55 ± 0.41	1.81 ± 0.58	0.32	2.02 ± 0.44	0.02	0.01	0.30	1.72 ± 0.52	0.21
*Tei (mm)*	1.80 ± 0.57	1.55 ± 0.42	2.09 ± 0.60	0.07	2.74 ± 0.59	< 0.001	< 0.001	0.01	2.51 ± 0.72	0.98
*Tei,max (mm)*	1.96 ± 0.73	1.49 ± 0.40	2.50 ± 0.65	0.003	4.00 ± 0.78	< 0.001	< 0.001	< 0.001	3.94 ± 0.83	0.84
*TFmax (%)*	17 ± 30	-3.5 ± 15	40 ± 24	0.001	100 ± 39	< 0.001	< 0.001	< 0.001	136 ± 41	0.07
**CT scan**										
*Right dome higher, n (%)*	14 (93)	7 (88)	7 (100)	1.0	19 (68)	0.13	0.52	0.21		
*L1 crus thickness (mm)*	3.6 [2.6; 8.8]	2.6 [1.5; 3.2]	9.4 [6.2; 12.1]	0.006	7.4 [4.7; 8.8]	0.13	< 0.001	0.17	5.0 [3.0; 7.1]	0.09
*Celiac crus thickness (mm)*	2.8 [2.2; 5.2]	2.2 [1.7; 2.6]	5.4 [4.2; 6.4]	0.008	3.8 [2.8; 2.6]	0.43	0.004	0.07	3.0 [2.5; 3.6]	0.39
*Height difference (cm)*	7.70 [4.68; 8.42]	8.08 [7.42; 8.73]	4.91 [3.94; 6.84]	0.13	1.16 [0.52; 1.73]	< 0.001	< 0.001	< 0.001		
Left dysfunction										
**N (%)**	24 (37)	17 (26)	7 (11)		28 (43)				22 (34)	
**Ultrasound**										
*Amplitude during QB (cm)*	0.00 [0.00; 0.85]	0.00 [0.00; 0.00]	1.20 [1.15; 1.30]	< 0.001	2.05 [1.62; 2.67]	< 0.001	< 0.001	< 0.001	3.00 [2.45; 3.40]	< 0.001
*Amplitude during DB (cm)*	-0.40 [-0.56; 1.85]	-0.51 [-0.65; -0.40]	2.30 [1.85; 2.55]	< 0.001	5.62 [5.00; 6.56]	< 0.001	< 0.001	< 0.001	5.40 [4.87; 6.21]	0.74
*Amplitude during VS (cm)*	-1.00 [-1.30; 1.00]	-1.23 [-1.41; -0.99]	1.40 [1.05; 1.60]	< 0.001	2.36 [1.98; 2.81]	< 0.001	< 0.001	< 0.001	2.90 [2.40; 3.78]	0.14
*Tee (mm)*	1.54 ± 0.35	1.48 ± 0.38	1.69 ± 0.24	0.19	1.91 ± 0.42	0.001	0.001	0.18	1.91 ± 0.36	0.34
*Tei (mm)*	1.57 ± 0.45	1.39 ± 0.40	1.96 ± 0.29	0.003	2.51 ± 0.62	< 0.001	< 0.001	0.03	2.82 ± 0.73	0.66
*Tei,max (mm)*	1.61 ± 0.58	1.35 ± 0.47	2.20 ± 0.29	< 0.001	4.00 ± 0.85	< 0.001	< 0.001	< 0.001	4.02 ± 1.04	0.94
*TFmax (%)*	5 ± 24	-6 ± 20	31 ± 7	< 0.001	112 ± 37	< 0.001	< 0.001	< 0.001	111 ± 41	0.40
**CT scan**										
*Left dome higher, n (%)*	22 (92)	16 (94)	6 (86)	1.0	9 (32)	< 0.001	< 0.001	0.03		
*L1 crus thickness (mm)*	2.0 [1.6; 2.7]	2.0 [1.6; 2.3]	3.5 [2.6; 4.3]	0.03	7.4 [4.6; 9.0]	< 0.001	< 0.001	0.005	4.7 [4.0; 5.7]	0.01
*Celiac crus thickness (mm)*	1.7 [1.4; 2.2]	1.8 [1.3; 2.2]	1.6 [1.6; 2.1]	0.80	3.1 [2.7; 3.8]	< 0.001	< 0.001	0.001	3.4 [3.0; 4.2]	0.61
*Height difference (cm)*	5.51 [2.77; 7.74]	6.70 [3.00; 8.00]	3.51 [1.85; 5.58]	0.09	1.16 [0.52; 1.73]	< 0.001	< 0.001	0.02		

*QB* quiet breathing, *DB* deep breathing, *DD* diaphragm dysfunction, *VS* voluntary sniffing, *Tee* thickness at end-expiration, *Tei* thickness at end-inspiration, *Tei, max* thickness at the end of maximal inspiration, *TFmax* maximal thickening fraction, *Contral* contralateral, *Para* paralysis, *Weak* weakness

In Figures S1, the correlations between ultrasound and CT measurements on the right and left sides are depicted. For both right and left diaphragmatic measurements, there was a robust correlation between CT measurements taken at the level of L1 and the celiac artery, with correlation coefficients of *r* = 0.71 (*p* < 0.001) and *r* = 0.74 (*p* < 0.001), respectively. On the right side, L1 measurements correlated significantly with amplitude during DP (*r* = 0.34, *p* < 0.01), during VS (*r* = 0.31, *p* < 0.05), and expiratory thickness (*r* = 0.26, *p* < 0.05). Additionally, a significant correlation was observed between measurements at the celiac artery and amplitude during QB (*r* = 0.32, *p* < 0.05). On the left, both L1 and celiac artery measurements correlated moderately to strongly with all ultrasound measurements (*p* < 0.001).

Table 3 presents the threshold values and diagnostic performances for DD of CT measurements derived from ROC curve analyses. The diagnostic performances of L1 and celiac artery measurements were found to be good in the case of paralysis, with better performance observed in left DD compared to right DD. However, in the case of weakness, the diagnostic performances of L1 and celiac artery measurements were good for left DD but not right DD with 95% confidence intervals for areas under the curve including 50%. The diagnostic performances of diaphragmatic cupola height differences were excellent, with areas under the curve close to 100%, except for left weaknesses, where the area under the curve was 79%.

**Table 3:** ROC curve analyses

Group	Measurement	Side	AUC	95%CI	Threshold	Accuracy	Se (%)	Spe (%)	PPV (%)	NPV (%)
Paralysis	L1 (mm)	R	0.93	84–100	4.5	0.87	87	87	95	70
	Celiac (mm)	R	0.84	70–98	3.0	0.75	71	87	94	50
	L1 (mm)	L	0.99	95–100	3.8	0.93	92	94	96	89
	Celiac (mm)	L	0.97	93–100	2.6	0.95	100	88	92	100
Weakness	L1 (mm)	R	0.67	43–92	9.4	0.81	57	87	57	87
	Celiac (mm)	R	0.73	49–96	4.9	0.84	71	84	62	91
	L1 (mm)	L	0.88	75–100	5.0	0.77	71	100	100	46
	Celiac (mm)	L	0.93	85–100	2.6	0.87	87	86	95	67
Paralysis	Height difference (cm)	R	1.00	100–100	4.4	1.00	100	100	100	100
Paralysis	Height difference (cm)	L	0.98	94–100	2.7	0.95	88	100	100	93
Weakness	Height difference (cm)	R	1.00	100–100	3.5	1.00	100	100	100	100
Weakness	Height difference (cm)	L	0.79	56–100	2.5	0.88	71	93	71	93

*R* Right, *L* Left, *AUC* area under the curve, *95%CI* 95% confident interval, *Se* sensitivity, *Spe* specificity, *PPV* positive predictive value, *NPV* negative predictive value

Threshold values of CT-scan measurements to diagnose diaphragm dysfunction (ultrasound as reference) at the level of the celiac artery or L1 (mm) and of the height difference (cm)

## Discussion

This study demonstrates a noteworthy reduction in diaphragmatic pillar thickness at the level of L1 and the celiac artery in the majority of cases, except for right weaknesses. Additionally, CT examination revealed a significant increase in diaphragmatic cupola height differences, regardless of the side or type (paralysis or weakness) of DD.

The absence of diaphragmatic pillar thinning in right-sided diaphragmatic weakness may be attributed to various factors. Among the 7 patients with right-sided diaphragmatic weakness, only 4 exhibited severe weakness, defined as greater muscle dysfunction with less than 40% of maximal thickening on ultrasound, whereas all weaknesses observed on the left were severe. Indeed, the mean thickening fraction in the case of weakness was 40% on the right, compared to 31% on the left. Additionally, the right diaphragmatic pillar is inherently stronger, thicker, and longer than the left pillar [10, 19]. It can be hypothesized that the right pillar that the change in the right pillar is less pronounced in the presence of simple diaphragmatic weakness.

Regarding diagnostic thresholds for diaphragmatic pillars thickness on CT, for right-sided diaphragmatic paralysis, ROC analysis identified a threshold of 3.0 mm at the level of the celiac artery and a threshold of 4.5 mm at the level of L1, both demonstrating good diagnostic performances [20]. Both thresholds exhibited good sensitivity, specificity, and PPV. Notably, the pillar measurement at the L1 level showed higher sensitivity and a higher AUC, with a significantly higher NPV. Therefore, for diagnosing right diaphragmatic paralysis, the pillar measurement at the L1 level appears to be more relevant than at the celiac artery level. For left paralysis, ROC analysis determined a threshold of 2.6 mm for the diaphragmatic pillar at the level of the celiac artery. This threshold remained identical for the left diaphragmatic weaknesses, as no significant difference in pillar thickness was found between the paralysis and weakness groups at this level. Moreover, at the L1 level, weakness can be differentiated from left diaphragmatic paralysis. The threshold for paralysis at L1 was 3.8 mm, exhibiting good diagnostic performance. For left diaphragmatic weakness, ROC analysis established a threshold of 5.0 mm at the L1 level with excellent diagnostic performance (specificity 100% and PPV 100%), ensuring a certain diagnosis of left diaphragmatic weakness if the thickness falls below the threshold.

The study conducted by Sukkasem et al. identified a threshold of 2.5 mm for diagnosing diaphragmatic paralysis on both the right and left sides, at the level of the celiac artery and L1 [10]. It's important to note that in their study, diaphragmatic pillar thickness was measured in patients with diaphragmatic paralysis compared to those with normal diaphragmatic function, and diaphragmatic function was assessed using fluoroscopy as the gold standard. Comparing the results of these two studies is challenging due to the use of different gold standards. Additionally, our study found larger right crus in the context of right weakness compared to controls and data reported by Sukkasem et al. It's suggested that ultrasound may be more sensitive than fluroroscopy and could potentially differentiate weakness from paralysis more precisely. A postulation is made that weakness might have a distinct pathophysiology compared to paralysis. Weakness may be associated with a loss of function without significant structural changes, resulting in muscle relaxation and thickening as fibers overlap.

In the DD group, the contralateral diaphragm exhibited compensation through an increase in amplitude during QB and VS in both right and left DD as observed in ultrasound. This increase in the amplitude of the healthy contralateral diaphragm has been previously documented as a neuronal compensation mechanism for the function of the contralateral hemi diaphragm in paralysis [12, 21, 22]. However, it is noteworthy that there was no evidence of hypertrophy in either the right or left healthy diaphragmatic pillar. Furthermore, CT scans revealed thinner pillars at the L1 level on the healthy side of patients with DD compared to controls (p = 0.01 on the right and p = 0.09 on the left), likely attributable to overall muscle weakness.

In the case of right diaphragmatic paralysis or weakness, ROC curve analysis determined a threshold of height difference between the two domes of 4.4 cm and 3.5 cm respectively, with perfect diagnostic performances of 100% [20]. This implies that the diagnosis of right diaphragmatic paralysis or weakness is certain if the height exceeds the respective threshold. For left DD, diagnostic performances were good for paralysis but not for weakness, with close values of height difference between the two domes (2.7 cm and 2.5 cm, respectively). Therefore, in our study, the height difference between the domes emerges as the most reliable CT measurement for diagnosing DD, potentially because diaphragm function may be compromised even in the absence of structural changes such as muscle atrophy. Notably, this study represents the first instance of precise threshold values for the diagnosis of DD based on the difference in cupola height being reported.

DD is typically linked to a modest reduction in vital capacity, around 75%, while TLC is generally maintained [23]. The more pronounced decrease in volumes observed in the weakness group in this study may be attributed to factors other than DD itself. Among the 12 patients exhibiting a restrictive pattern in the weakness group, several had conditions potentially influencing lung function. This includes 6 patients with a history of COVID-19 (5 of whom experienced severe diseases requiring intensive care), one patient with a history of talc pleurisy, 2 patients who were overweight, and 6 patients who were moderately obese.

This work is subject to several limitations. First, CT has certain drawbacks in comparison to ultrasound, including impracticality at the bedside, higher cost, and exposure to radiation. Nevertheless, CT is routinely employed in clinical practice, particularly in the assessment of dyspnea. Despite its inherent disadvantages relative to ultrasound, CT is almost systematically utilized in the evaluation of diaphragm dysfunction due to its widespread availability and comprehensive lung imaging capabilities.

Second, it is acknowledged that pillar thickness is expected to vary with breathing, decreasing with expiration [24]. In this study, only one radiologist conducted the measurements, and the intra- and inter-reproducibility of these measurements were not assessed). Additionally, both enhanced and unenhanced CT scans were utilized, which could pose challenges in measuring the pillar thickness in unenhanced CT. If the scan is performed during expiration, there is a risk of mistakenly assuming DD. Moreover, in our study, to ensure correct inspiration during thoracic scans, we verified that the posterior part of the trachea did not inwardly bulge. Howevern this check could not be performed for the single abdominal scan included in our study.

Third, CT were conducted during the inspiratory phase, while ultrasound examinations were performed during the expiratory phase. Despite the disparate conditions associated with these two imaging modalities, both methods were systematically executed within identical respiratory phase. However, it is important to acknowledge that this difference in respiratory phases could introduce variability in the measurements and should be considered when interpreting the results. The observed correlations between measurements indicate a degree of association despite the differences in imaging timing, but this discrepancy should be kept in mind when extrapolating findings from one modality to the other.

Fourth, the scans were performed at different times, either before or after the ultrasound, with most instances occurring before ultrasound (median time between CT and ultrasound was -20 days in DD). Since the structure of the diaphragm can change during illness, the time gap between the two examinations may differently affect pillar atrophy. However, both imaging modalities were performed after the appearance of DD. The exam conducted later in the course of DD may be influenced by a recovery process, potentially attenuating the correlation between measurements. Given that ultrasound is considered the gold standard in this study, if CT was performed before ultrasound, diaphragm measurements on CT would be reliably reflective of the DD condition. However, if performed after ultrasound, CT measurements might be underestimated due to the ongoing recovery process.

Fifth, it is important to note that there were more women in the paralysis group than in the weakness group (*p* < 0.001). It can be assumed that diaphragmatic pillars are thinner in women, as seen in ultrasound. However, it is worth mentioning that in the study by Dovgan et al. [25], pillar thickness did not vary according to gender. This inconsistency highlights the potential impact of gender distribution on the results and emphasizes the need for further investigation and clarification regarding the relationship between gender and diaphragmatic pillar thickness.

## Conclusion

This study revealed a noteworthy reduction in diaphragmatic pillar thickness at the level of L1 and the celiac artery on CT in cases of DD, observed in both paralysis and weakness on the left, and exclusively in paralysis on the right side. In both right and left DD, a significant difference in height between the two diaphragm domes was noted in cases of diaphragmatic paralysis or weakness, demonstrating good diagnostic performances.

The study identified the measurement with the most favorable diagnostic performance for right paralysis or weakness as the difference in height between the two domes, with thresholds of 4.4 cm and 2.5 cm, respectively. On the left side, for paralysis, diagnostic performance of the measurement at L1 and the difference in height between the two domes were equivalent (thresholds of 3.8 mm and 2.7 cm, respectively). For weakness on the left, the measurement with the best diagnostic performance was the thickness at the L1 level, with a threshold of 5.0 mm).

### Supplementary Information

**Additional file 1: Figure S1.** Spearman’s correlation matrix between ultrasound and CT scan measurements. A, Correlation between ultrasound and CT scan measurements on the right side of the 65 patients included in the study. B, Correlation between ultrasound and CT scan measurements on the left side of the 65 patients included in the study. L1 = pillar thickness at the level of L1 on CT scan, Celiac = pillar thickness at the level of the celiac artery on CT scan, Amp QB = amplitude during quiet breathing, Amp DB = amplitude during deep breathing, Amp VS = amplitude during voluntary sniffing, Tee = thickness at end-expiration, Tei = thickness at end-inspiration, Tei max = = thickness at the end of maximal inspiration, TF max = maximal thickening fraction. Each significance level is associated with a symbol: *** for *p *<0.001, ** for *p *<0.01, * for *p *<0.05, square dot for *p *<0.10

## Data Availability

No datasets were generated or analysed during the current study.

## Abbreviations

BMI: Body mass index
CT: Computed tomography
DB: Deep breathing
DD: Diaphragmatic dysfunction
QB: Quiet breathing
TF_max_: Thickening fraction
VS: Voluntary sniffing
