# Supplementary material for: Evaluation of computed tomography in the diagnosis of ultrasound-proven diaphragm dysfunction
Source: Respir Res. 2024 Mar 20;25:135. doi: 10.1186/s12931-024-02770-w (PMC10956268; doi:10.1186/s12931-024-02770-w)
Supplement: Supplementary file 1 — Additional file 1: Figure S1. Spearman’s correlation matrix between ultrasound and CT scan measurements. A, Correlation between ultrasound and CT scan measurements on the right side of the 65 patients included in the study. B, Correlation between ultrasound and CT scan measurements on the left side of the 65 patients included in the study. L1 = pillar thickness at the level of L1 on CT scan, Celiac = pillar thickness at the level of the celiac artery on CT scan, Amp QB = amplitude during quiet breathing, Amp DB = amplitude during deep breathing, Amp VS = amplitude during voluntary sniffing, Tee = thickness at end-expiration, Tei = thickness at end-inspiration, Tei max = = thickness at the end of maximal inspiration, TF max = maximal thickening fraction. Each significance level is associated with a symbol: *** for p <0.001, ** for p <0.01, * for p <0.05, square dot for p <0.10 [file 12931_2024_2770_MOESM1_ESM.pdf]

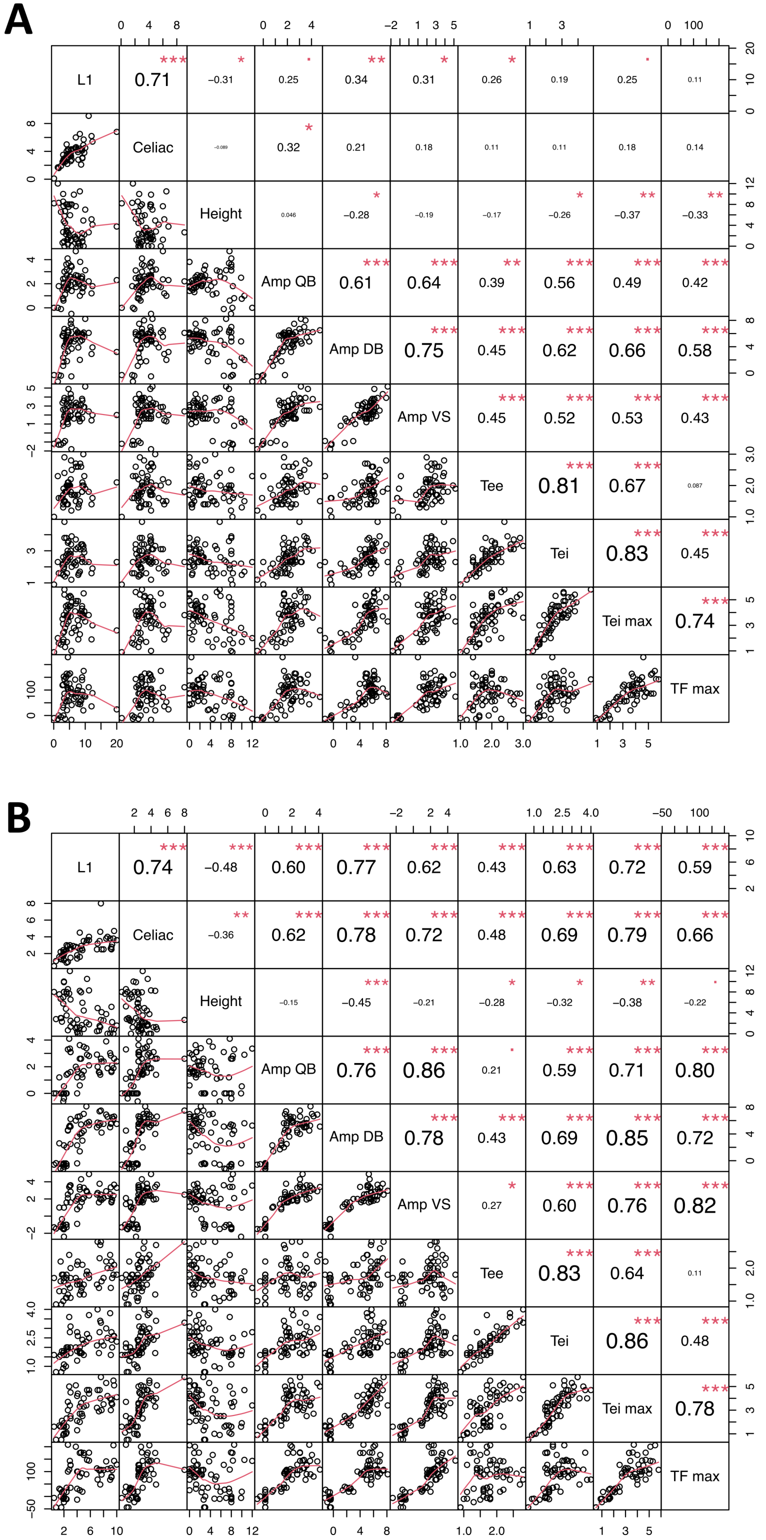
A
L1
0.71
−0.31
0.25
0.34
0.31
0.26
0.19
0.25
0.11
Celiac
0.32
0.21
0.18
0.11
0.11
0.18
0.14
Height
−0.28
−0.19
−0.17
−0.26
−0.37
−0.33
Amp QB
0.61
0.64
0.39
0.56
0.49
0.42
Amp DB
0.75
0.45
0.62
0.66
0.58
Amp VS
0.45
0.52
0.53
0.43
Tee
0.81
0.67
Tei
0.83
0.45
Tei max
0.74
TF max
B
L1
0.74
−0.48
0.60
0.77
0.62
0.43
0.63
0.72
0.59
Celiac
−0.36
0.62
0.78
0.72
0.48
0.69
0.79
0.66
Height
−0.15
−0.45
−0.21
−0.28
−0.32
−0.38
−0.22
Amp QB
0.76
0.86
0.21
0.59
0.71
0.80
Amp DB
0.78
0.43
0.69
0.85
0.72
Amp VS
0.27
0.60
0.76
0.82
Tee
0.83
0.64
0.11
Tei
0.86
0.48
Tei max
0.78
TF max
